# The health, financial and distributional consequences of increases in the tobacco excise tax among smokers in Lebanon

**DOI:** 10.1016/j.socscimed.2016.10.020

**Published:** 2016-12

**Authors:** Nisreen Salti, Elizabeth Brouwer, Stéphane Verguet

**Affiliations:** a Department of Economics, American University of Beirut, Beirut, Lebanon; b Department of Global Health, University of Washington, Seattle, WA, USA; c Department of Global Health and Population, Harvard T.H. Chan School of Public Health, Boston, MA, USA

**Keywords:** Lebanon, Tobacco taxation, Equity, Financial risk protection, Distributional consequences, Extended cost-effectiveness analysis

## Abstract

Tobacco use is a significant risk factor for the leading causes of death worldwide, including cancer, heart disease and stroke. Most of these deaths occur in low- and middle-income countries, where tobacco-related deaths are also rising rapidly. Taxation is one of the most effective tobacco control measures, yet evidence on the distributional impact of tobacco taxation in low- and middle-income countries remains scant. This paper considers the financial and health effects, by socio-economic class, of increasing tobacco taxes in Lebanon, a middle-income country.

An Almost Ideal Demand System is used to estimate price elasticities of demand for tobacco products. Extended cost-effectiveness analysis (ECEA) methods are applied to quantify, across quintiles of socio-economic status, the health benefits gained, the additional tax revenues raised, and the net financial consequences for households from a 50% increase in the price of tobacco through excise taxes. We find that demand for tobacco is price inelastic with elasticities ranging from −0.32 for the poorest quintile to −0.22 for the richest quintile. The increase in tobacco tax is estimated to result in 65,000 (95% CI: 37,000–93,000) premature deaths averted, 25% of them in the poorest quintile, $300M ($256–340M) of additional tax revenues, 12% borne by the poorest quintile, $23M ($13–33M) of out-of-pocket spending on healthcare averted, 36% of which accrue to the poorest quintile, 9% to the richest. These savings would be associated with 23,000 (13,000–33,000) poverty cases averted (63% in the poorest quintile). Increasing tobacco taxes would lead to large financial and health benefits, and would be pro-poor in health gains, savings on healthcare, and poverty reduction.

## Highlights

• Health benefits from higher tobacco taxes would be progressive in Lebanon.
• Health benefits also result in health expenditures averted which are pro-poor.
• Tax revenue raised from higher tobacco taxes is borne mostly by richer quintiles.

## 1 Introduction

Non-communicable diseases (NCDs) are the leading cause of death worldwide, and the vast majority of NCD deaths now occur in low- and middle-income countries (Alwan et al., 2010, Murray et al., 2015). Half of NCD-related deaths occur during the prime productive years of adulthood, resulting in substantial societal costs that extend beyond health service delivery (Bloom et al., 2011).

Tobacco is a significant risk factor for NCDs including cardiovascular disease, cancer and stroke. The World Health Organization (WHO) puts an estimate of the annual economic burden of tobacco-related illnesses at over $500 billion, which exceeds total annual health expenditures in low- and middle-income countries (WHO, 2014b). Without significant intervention, the number of tobacco-related deaths in low- and middle-income countries is projected to reach 7 million deaths per year by 2030, doubling the level of 2010 (NCD Alliance, 2011).

As a middle-income country, Lebanon is no exception to these trends. NCDs are the country's main killer, with ischemic heart disease alone accounting for over 30% of all deaths (IHME, 2013). Lebanon's disease burden is undoubtedly related to its smoking prevalence and intensity, which are among the highest in the Middle East and the highest for women in the Arab world (Salti et al., 2014, WHO, 2013). Average smoking prevalence rates are around 43% for men and 28% for women, and these rates have been consistently rising for decades (Sibai and Hwalla, 2010). Tobacco consumption increased by an alarming 475% between 1990 and 2012, which ultimately put annual consumption of cigarettes at 2400 per capita, three times the world average (Al-Akhbar English, 2013). In 2008, studies estimated that tobacco consumption cost the Lebanese economy an annual minimum of $325 million, close to 1% of the country's gross domestic product (GDP) in that year (Salti et al., 2014). We estimate that total spending on tobacco products in the Lebanese market is even larger at $850 million (CAS, 2005, National Customs Authority of Lebanon, 2012), just under 2% of GDP.

Despite tobacco's negative impact on both population health and the economy, the Lebanese government has not fully used the policy tools at its disposal to stem the epidemic. In 2011, pursuant to its ratification of the Framework Convention on Tobacco Control in 2005, the Lebanese parliament passed Law 174 to control the consumption of tobacco products. Specifically, the law prohibits smoking in indoor public spaces, bans advertising of tobacco products, and mandates the inclusion of text and pictorial warnings on tobacco packaging. Unfortunately, the enforcement of the law has been patchy at best, particularly in the area of the control of smoking in public places (Al-Akhbar English, 2014). Tobacco tax rates in Lebanon are also suboptimal at about 47% of the retail price for imported cigarettes; the World Health Organization recommends tobacco taxes be at least 70% of the retail price (WHO, 2010). Tobacco products are also comparatively affordable in Lebanon. Using the fraction of GDP per capita required to purchase 100 packs of the most sold brand of cigarettes as an indicator, tobacco products are more affordable in Lebanon than in neighboring or regional countries, including Jordan, Egypt, Turkey, or the West Bank and Gaza (WHO, 2015).

Some studies have looked at the consumption and revenue effects of raising tobacco taxes in Lebanon (Salti et al., 2015) and other LMICs (Levy et al., 2006, Blecher, 2011a), however these analyses fail to capture broader economic and health benefits. In this paper, we conduct an extended cost-effectiveness analysis (ECEA) (Verguet et al., 2015a, Verguet et al., 2015b, Verguet et al., 2015c) to examine the distributional consequences and household financial and health benefits (per socio-economic group) of a hypothetical increase in the excise tax on tobacco in Lebanon.

## 2 Methods

ECEA methods are described in Verguet et al. (2015b), and particularly in the context of tobacco tax in Verguet et al. (2015a). Health policy instruments such as public finance or taxation of tobacco products entail consequences in multiple domains. Fundamentally, they aim at leading to better health benefits (e.g. mortality averted), but these policies can also provide non-health benefits. For instance, tobacco taxes can prevent illness-related impoverishment and provide financial risk protection. Furthermore, they can improve the distribution of health in the population. ECEA is meant to evaluate the health and financial consequences of policies in the following three domains: the health gains, the financial risk protection benefits, and the distributional (e.g. across socio-economic groups) benefits. In this study, we draw closely on the approach used by Verguet et al. (2015a) and conduct an ECEA to examine the household health and financial benefits, and overall distributional consequences of increasing the tobacco excise tax in Lebanon.

First, we identify the price elasticities by age and income groups. We simulate the effect of an increase in the tobacco excise tax on: i) the change in out-of-pocket (OOP) expenditures on cigarettes, ii) the change in government revenue, iii) premature deaths averted, iv) the change in OOP expenditures on tobacco-related diseases, and v) associated poverty cases averted. All of these outcomes are estimated for the current population of smokers. We then use sensitivity analysis to test our findings with regards to potential substitute tobacco products. In Appendix 5 of the supplementary materials, we also translate the result on premature deaths averted into life years gained.

### 2.1 Group-based price elasticities

The price of most tobacco products is regulated by the *Régie*, the state-run monopoly in charge of regulating the market for tobacco, and the Ministry of Finance (Tobacco Fact in Lebanon, (2001)). Using $2.15 as the average price of a pack of imported cigarettes (Mahdi, 2014), we estimate the effect on current smokers of an increase in the excise tax that results in a 50% increase in the retail price of imported cigarettes. The analysis focuses solely on imported cigarettes as they represented about 90% of household spending on tobacco in 2005, and 75% of total cigarettes smoked (Chaaban et al., 2010).

The magnitude of the price increase of 50% is chosen because it would be a politically feasible change in tax. Because, as stated above, the *Régie* sets the retail price, the excise tax, and the profit margins of the distributors and retailers, the *Régie* also effectively controls the pass-through rates. At the current ratio of taxes to price for imported cigarettes (which is 47%), for instance, the Régie could decide to increase the price by 50% by increasing taxes. At the average price of $2.15 per pack, if the price increase of $1.075 were collected in additional taxes, the resulting tax would be closer to 65% of the new price. In the sensitivity analysis reported in Appendix 4 of the supplementary materials, we also look at the outcomes under different scenarios of price increases.

We use data from the Ministry of Public Health from 2011 to summarize population size and the relative sizes of age cohorts, focusing on individuals 15 and older (Ministry of Public Health of Lebanon (2011)). We have prevalence data by 10-year age groups (IHME, 2013, Sibai and Hwalla, 2010, Global Youth Tobacco Survey Country Factsheet for Lebanon, 2011) and by income quintile (National Household Health Expenditure and Use Survey, 1999). In order to obtain prevalence figures by both quintile and age group, we use prevalence by age group to calculate the total number of smokers in each age group. For each age group, we then allocate these smokers to quintiles by assuming that the distribution of smokers across quintiles is the same for each age group.

Demand elasticity for tobacco is estimated using primary data on household consumption from a nationally representative survey from 2005 (CAS, 2005), and an Almost Ideal Demand System (AIDS). The methodology is described in detail in Deaton and Muellbauer (1980) and Deaton (1990). We use spatial variation in relative prices to estimate elasticities: the Central Administration for Statistics releases price indexes for each of a number of consumables by district. Price variation in the AIDS model comes from geographical differences in these price indexes (Deaton, 1990). The AIDS model consists in running a constrained regression of the share of imported cigarettes in total household expenditures on a vector of prices. Elasticities are computed as nonlinear functions of the regression coefficients. The standard errors of the elasticities are then calculated using the delta method (by taking a first order Taylor series approximation) (Hosmer et al., 2008). These elasticities are estimated separately for each quintile. Appendix 7 of the supplementary materials shows the detailed regression results that yield these elasticity estimates. Quintiles are defined using household annual expenditures per adult equivalent, using data from the same household survey (CAS, 2005). The cutoffs for quintiles are reported in Table 1.

The results, with elasticities ranging between −0.32 and −0.22 over the five quintiles, are in line with the elasticity of demand for imported cigarettes estimated at −0.22 in Salti et al. (2015). While we are able to use our AIDS model to estimate demand elasticities by quintile, we do not have the data needed to estimate elasticity by age group or by gender. We assume that the elasticity for those under 24-years old is twice as large as the elasticity calculated for the whole population, which is consistent with the evidence reviewed by IARC (IARC, 2011), the WHO (2010) and the Asian Development Bank (2012). In the same vein, Levy et al. (2006) find higher elasticities of demand for youth in Vietnam, and Salti et al. (2015) find that demand for tobacco by households with younger heads is more elastic. Quintile-based elasticities obtained from the AIDS model are applied to all age groups above 25, and doubled for the younger age group.

### 2.2 Out-of-pocket tobacco expenditures and change in government revenue

Starting with current expenditures on tobacco by quintile, we use these estimated quintile-based elasticities to calculate the effect of a price change on each quintile's expenditures on tobacco (CAS, 2005, National Customs Authority of Lebanon, 2012). The tax will induce some smokers to quit, so these additional expenditures on tobacco are borne by continuing smokers in each quintile. Estimating changes in OOP expenditures on tobacco by consumers also allows us to estimate the change in taxes paid by each quintile. We are thereby able to calculate the change in tax revenue to the government.

To look at distributional consequences, after dividing the results by quintile population to get per capita figures, we scale the findings to the level of the average total household expenditures per adult equivalent in each quintile. This allows us to report in Table 3 the change in tobacco expenditures as a share of total household expenditures per adult equivalent for each quintile.

**Fig. 1:**
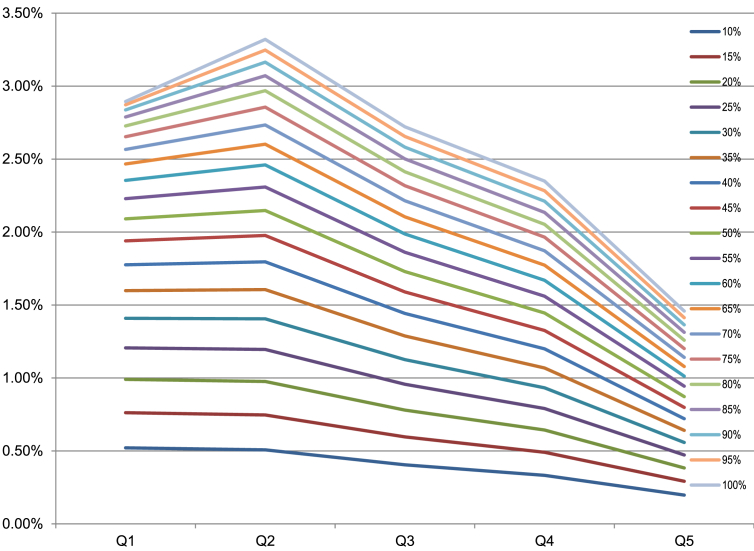
Share of expenditures on imported cigarettes in total household expenditures by quintile for different price changes.

We check the robustness of our estimates of the distributional consequences of this change in expenditures by looking in Fig. 1 at the effects on this relative share of household expenditures on tobacco of different magnitudes of price increases, ranging from 10% to 100%.

### 2.3 Premature deaths averted

To calculate deaths averted from an increase in the price of cigarettes, we assume that half of the price elasticity of demand estimated is an elasticity of participation, a standard assumption in the literature based on findings in several countries (WHO, 2010, Verguet et al., 2015a, IARC, 2011, Lewit and Coate, 1982, Mullahy, 1985, Wasserman et al., 1991, Evans and Farrelly, 1998). In the case of Lebanon, this participation elasticity for smoking is calculated as half the price elasticity of demand for imported cigarettes, since these account for an overwhelmingly large share of the market, as mentioned above. Quitters for each age-quintile group are then directly calculated from the participation elasticity and the increase in cigarette price that results from the tax. The price change would also result in reduced tobacco consumption among continuing smokers, but we do not include the health benefits for this reduced intensity of smoking in our estimates of health gains. Only deaths averted from quitting are taken into consideration in our estimation of health benefits. Doll et al. (2004) estimates of the effect on the reduced relative risk of death by age at quitting were used (Table 1). Deaths averted by quitting for each age group are estimated here as follows: we use a 50-year time frame and assume that over the next 50 years, half of smokers die of their habit (Doll et al., 2004). More recent findings on cohorts aged 45 and above find that as many as two thirds of smokers die from smoking (Banks et al., 2015), however, we use the more conservative estimates. We apply this schedule of reduced mortality risk to our estimated quitters (which differ by age and quintile because of different prevalence and elasticity by quintile-age group).

### 2.4 Health system and out-of-pocket costs averted

While there are clear immediate and long-term health benefits from quitting smoking, from a lifetime perspective, smoking cessation may also be associated with some healthcare costs and not just healthcare savings, as the years of life gained from quitting smoking may come with healthcare costs at advanced ages. However, work by Hodgson (1992), which compares lifetime healthcare costs for smokers and non-smokers, shows that smokers incur higher total lifetime healthcare costs. An updated estimation of the Hodgson results (Boonn, 2014) using 2009 data, and applying the Centers for Disease Control estimates of the cessation-association relative reduction in the risk of death, finds that quitting is associated with total lifetime healthcare savings. Similarly, work by Fishman et al. (2003), and by Rasmussen et al. (2005) also shows that smoking cessation is associated with a reduction in healthcare costs for quitters, even in the long run. In this study, we do not take into account any healthcare costs incurred by quitters in the years of life gained. We focus instead on estimating the savings for the health system and for the individual quitter associated with the deaths averted from quitting, keeping in mind the above cited evidence that quitting is associated with long-run healthcare savings for both the system and the individual. We limit our calculations of health spending averted to savings on hospitalization costs.

There are 2 main statistics needed to calculate hospitalization costs saved by quintile due to the deaths averted: the cost of tobacco-related hospitalizations and the utilization rate of healthcare services by quintile. We detail the estimation of each of these two measures in turn.

We consider only five disease groups associated with tobacco consumption in our calculations of hospitalization costs saved due to deaths averted. These disease groups are cardiovascular disease, stroke, chronic obstructive pulmonary disorder (COPD), lung and bladder cancers. These diseases combined account for the majority of tobacco deaths, and data on these diseases is available from the Global Burden of Disease (GBD) study (IHME, 2013). We use hospitalization costs for each of the five diseases (Karam, 2014) and data on the distribution of tobacco-related deaths across these causes of death (IHME, 2013) to estimate the cost of a tobacco-related hospitalization.

Utilization rates by diagnosis are estimated as follows: the Ministry of Public Health provides data by diagnosis on hospitalizations covered by the ministry (Ministry of Public Health, 2011). These data, in conjunction with the fact that the Ministry of Public Health covers on average 12% of hospitalizations (UNDP, 1997), are used to obtain total annual hospitalizations for these diagnosis groups. As detailed in Appendix 1 of the supplementary materials, we calculate utilization rates by comparing these imputed total hospitalizations per year for each diagnosis to the prevalence rates of the five disease groups (WHO, 2014a, Jurjus et al., 2009, American Lung Association,, Schneider et al., 2007, American Cancer Society,). We thus obtain disease-specific utilization rates for the whole income scale.

In order to estimate hospitalizations by quintile, we need quintile-based utilization rates. From the NHHEUS, we have data on utilization rates by quintile for any healthcare service, conditional on having a health condition (National Household Health Expenditure and Use Survey, 1999). These utilization rates are normalized using the middle quintile as a base and scaling the utilization rates of other quintiles as relative utilization compared to the middle quintile. We apply to the disease-specific utilization rates the relative utilization rates of hospitalizations by quintile to obtain the utilization rate of hospitalization services by disease/quintile group. The health system savings on hospitalization costs are calculated for each quintile by multiplying the deaths averted by the average cost of a smoking-related hospitalization and the utilization rates by quintile and by disease. Subsequently, we derive the savings in OOP health expenditures using the share of healthcare costs paid out of pocket by expenditure quintile, reported in Table 1 (Salti et al., 2010). OOP health expenditures averted are calculated keeping in mind that insurance plans cover acute health expenditures associated with hospitalizations due to tobacco-related illnesses, and patients without insurance coverage pay out of pocket. We assume that quitters in each quintile have the same insurance coverage rate as the overall quintile. In each quintile, we estimate OOP expenditures by applying to the hospitalization costs incurred by the quintile (described above) the insurance coverage rate of the same quintile.

### 2.5 Cases of poverty averted

OOP health expenditures averted by quintile are then used to estimate the number of poverty cases averted. The reasoning here is that for some quitters, money they would have spent on healthcare is now made available to spend on consumption. We assume that these health savings raise expenditures, and therefore lift the individuals' position relative to the poverty line. The World Bank puts the poverty line for Lebanon at $4 of spending per person per day in 2008. Adjusted for inflation using World Bank estimates of the Consumer Price Index (CPI) and the Gross Domestic Product (GDP) deflator, the poverty line stands at $5.5 in 2012. The poverty headcount estimated by the Ministry of Social Affairs is 29% of the population and the poverty gap is 9% (International Poverty Center, 2008). With a poverty rate of 29%, all of the bottom quintile is below the poverty line, and 40% of the second quintile is poor. These figures are consistent with our findings for average household consumption per capita, which stand at below the poverty line for the lowest quintile and above it for the second quintile. We assume that deaths averted in the second quintile are uniformly distributed over the quintile, so that the incidence of poverty among quitters from the second quintile is the same as the incidence of poverty of the overall quintile (at 40%). So for the second quintile, in order to estimate the effect of the OOP health savings on poverty, we consider the 40% of quitters from the second quintile that fall below the poverty line before the tax increase. Because we do not know the effect on OOP health spending of reducing the intensity of smoking for continuing smokers, we do not estimate the effect of the tax on the poverty status of smokers who continue to consume tobacco after the tax. It is likely, therefore, that our analysis underestimates the true benefits of an increase in tobacco tax rate.

### 2.6 Sensitivity analysis

We run a number of sensitivity tests of our main results that allow us to check the sensitivity of our results to some of the simplifying assumptions made and to investigate the robustness of the main findings to extensions in the types of tobacco products consumed and the choice of parameters.

The results pertaining to the distribution of outcomes are obtained under a number of assumptions which we re-examine in turn: we first reconsider the assumption about the elasticity of younger consumers, and report the results in Appendix 2 of the supplementary materials, we then vary the assumption about the share of participation in the elasticity of demand for cigarettes and report the results in Appendix 3 of the supplementary materials.

We expand our analysis of the effect of tobacco taxes on tax revenues and household expenditures on tobacco by quintile to include three tobacco products: in addition to imported cigarettes, considered in the main analysis, we now also look at locally produced cigarettes and waterpipe (or hookah) tobacco. We use the same AIDS model to calculate own- and cross-price elasticities for all three tobacco products in Lebanon and we estimate these separately by expenditure quintile. We then estimate the resulting tax revenue and household expenditures on tobacco while taking into account possible substitutions across tobacco products. This extension is reported in Appendix 4 of the supplementary materials.

We also consider different scenarios for the increase in taxes, including a 25% and a 100% increase in retail price.

The AIDS model is run using STATA 12.0. All data on inputs are shown in Table 1.

## 3 Results

Table 2 shows point estimates for own-price elasticity of demand for imported cigarettes. It also shows 95% confidence intervals for the elasticities. Demand is inelastic for the entire range of the 95% confidence interval for each quintile. The point estimates of elasticities are monotonically increasing as we move from richer to poorer socio-economic groups, ranging from −0.32 (95% CI −0.47:−0.18) for the poorest quintile to −0.22 (−0.31:−0.14) for the richest. This inverse relationship between the sensitivity of demand and socio-economic status is in line with the findings in the literature. Regressions in the AIDS model are run separately for each quintile. Each elasticity estimate is based on a regression for 1045 households in that quintile. The full details of the regressions are reported in Appendix 7 of the supplementary materials.

Tobacco expenditures increase for all expenditure quintiles, however the magnitude of this increase is relative. When scaled to household expenditures per adult equivalent, this extra spending on tobacco products amounts to 2.1% (CI: 1.2%–2.9%) of total household expenditures per adult equivalent for the poorest expenditure quintile, and 0.9% (CI: 0.7%–1%) for the top quintile. This additional burden falls on continuing smokers in each quintile.

The distribution across expenditure quintiles of the added expenditures on cigarettes is different depending on the level of cigarette price increase. Fig. 1 shows the size of the increase in spending on cigarettes scaled to household expenditures per adult equivalent for each quintile, and tracks this ratio for different levels of price increases ranging from 10 to 100%. The figure shows that for relatively large increases in price, the effect on expenditures on cigarettes as a share of total household expenditures per adult equivalent peaks for the second poorest quintile. For price increases in the middle range (25%–45%), the effect on the poorest and second quintiles is similar, and larger than for the wealthier quintiles. For smaller price increase, the added share of household expenditures per adult equivalent devoted to cigarettes is highest for the poorest quintile and decreases monotonically over the income scale. These results are driven by the underlying distribution of household expenditures per adult equivalent as well as the quintile differences in smoking prevalence and elasticities.

A direct implication of the increased overall expenditures on tobacco is an increase in government revenue. We simulate a 50% increase in price that is entirely levied by the government, since the *Régie* regulates the tobacco market. The price increase would result in higher tax revenues by close to $300 million per year (95% CI: $256M-$340M), which represents an 80% (95% CI: 68%–91%) increase in tax revenues from imported cigarettes. Nearly 26% of the extra tax burden is financed by the richest quintile while only 12% is borne by the poorest.

In Table 3, we examined the premature deaths averted among current smokers over the course of their lifetime. A 50% increase in the price of tobacco would avert close to 65,000 premature deaths from among estimated quitters (95% CI: 37,000–93,000), over 25% of which accrue to the poorest expenditure quintile. The distribution of deaths averted is monotonically progressive with only 14% of deaths averted from the richest expenditure quintile. In the sensitivity analysis, we re-examine this result while loosening the assumption about a higher elasticity of demand for the youth. The deaths averted are, unsurprisingly, slightly lower when the elasticity is assumed to be age-invariant, ranging from 13,500 (95% CI: 7600–19,800) in the poorest quintile to 7500 (95% CI: 4700–10,500) in the richest, but the overall distribution of deaths averted remains progressive across all 5 quintiles. The magnitude of the findings on deaths averted is also sensitive to the assumption about the share of participation in the overall elasticity of demand, but again, the pattern observed on the distribution of deaths averted over quintiles is qualitatively robust to the fraction of elasticity attributed to participation (Appendix 3 of the supplementary materials).

The deaths averted are associated with savings on health spending estimated at almost $37 million (95% CI: 21M-53M), which amounts to close to $22 million in household OOP savings. Scaled to the total number of smokers, these figures represent around $41 of health care system savings per smoker per year, or around $26 of OOP savings per smoker per year. Of the household OOP savings, 36% accrue to the poorest quintile while only 9% are for the richest. These savings represent close to 0.6% (95% CI: 0.3%–0.9%) of household expenditures by adult equivalent for the poorest quintile. In appendix 6 of the supplementary materials, these savings are shown in net present value, discounted over 50 years (assuming they accrue for each cohort when the quitter reaches life expectancy) using a 3% per year discount rate. When we assume no difference between the elasticity of younger and older cohorts, these savings are slightly smaller in size, as expected, but their distribution across quintiles remains qualitatively unchanged both for total health system savings as well as OOP savings for households (Appendix 2 of the supplementary materials).

OOP healthcare spending on tobacco-related diseases can have an impoverishing effect and push households below the poverty line. We estimate the number of poverty cases averted from a 50% price increase on imported cigarettes at close to 27,000 individuals (13,000–32,000), 63% of whom would be in the poorest quintile. This represents 2.0% of all people in the poorest quintile and around 1.2% of people in the second poorest quintile. When the elasticities for young and old are assumed to be the same, the poverty implications of the tax increase are again qualitatively unchanged, as shown in Appendix 2 of the supplementary materials: even when health expenditures averted are smaller, they are sufficient per quitter to lift above the poverty line all quitters with a household consumption per capita equal to the quintile average for the lowest quintile. For the second quintile, if we maintain the assumption that the average household consumption spending per capita of quitters is uniformly distributed over the quintile, then the 40% of quitters who are below the poverty line before the tax benefit from sufficient health savings to push them out of poverty.

## 4 Discussion

Lebanon is currently one of the cheapest places to buy both imported and local cigarettes in the Arab region: as mentioned above, cigarettes are less affordable in Jordan, Egypt, Turkey, Cyprus, and the West Bank and Gaza (WHO, 2015). With taxes totaling only 47% of the price (Mahdi, 2014), there is hence substantial room to increase these taxes.

Our price elasticity findings fall on the more inelastic end of the range of estimates for other middle-income countries, with Egypt at −0.27 to −0.82 (Nassar, 2003), Turkey at −0.41 (Onder, 2002) and South Africa at −0.46 (Blecher, 2011b). These are also similar to the findings of Levy et al. for Vietnam (Levy et al., 2006). Other studies that use an AIDS approach find elasticities on the order of −0.53 for Vietnam (Eozenou and Fishburn, 2009).

Tobacco taxation is a well-established measure for decreasing tobacco consumption (Levy et al., 2006, Blecher, 2011a, Blecher, 2011b, IARC, 2011). While some studies have looked at the distributional impact of raising tobacco taxes in high-income settings (Colman and Remler, 2008, Warner, 2000, Chaloupka and Warner, 2000) and found mixed results, few studies have considered the distributional consequences and equity of such measures (Verguet et al., 2015a). This study adds to the literature by examining the effect of an increase in the excise tax on tobacco in Lebanon by quintile.

In this paper, we look at five outcomes, by expenditure quintile, of a 50% increase in tobacco price. We find that nearly 65,000 deaths are averted, over 25% of which are from the poorest quintile. Health gains are progressively distributed, with a larger advantage accruing to the poorer quintiles. We estimate that $37 million of health expenditures are averted, $22 million of them paid out of pocket by households. Of these, 36% are saved by the poorest quintile. Rehm et al. (2006) find that hospitalizations constitute 56% of total economic costs of tobacco in Canada. Using the same cost breakdown, our findings of $37 million in savings from averted hospitalizations would be associated with around $246 million in total economic savings, including productivity losses averted. Using findings from similar research in the UK (ASH, 2015) would put a lower bound on total economic savings associated with the averted hospitalizations at $270 million.

The health expenditures averted result in 17,000 cases of poverty averted in the poorest quintile. The total number of poverty cases averted is close to 27,000, around 2.3% of the poverty headcount.

The effects on health spending and the resulting poverty reduction are therefore also pro-poor. When we compare the fraction of deaths averted and the fraction of the total tax burden accruing to the lowest quintile, our results are in line with some of the findings in the literature: a study by the Asian Development Bank (2012) finds that the benefit to tax ratio for groups with the lowest socioeconomic status for a 50% price increase is around 1.4 for Vietnam, 1.5 for India and 1.9 for the Philippines. The ratio we obtain for Lebanon is 2.2.

Household expenditures on tobacco would increase by $245 million, 11% of which are spent by the poorest quintile and 27% by the richest. As a fraction of total household expenditures, however, the additional spending on tobacco is a larger share of household expenditures for poor quintiles (2.1%) than that of rich quintiles (0.9%). Tax revenues increase by close to $300 million. 12% of the additional tax burden is borne by the poorest quintile, and over 26% is financed by the richest quintile, financed entirely by continuing smokers in each quintile.

The distribution of additional expenditures on tobacco as a fraction of household expenditures is linked to several factors: the differences in the prevalence of smoking across quintiles, the differences in elasticities across quintiles, and the underlying degree of inequality in household expenditures across quintiles. The distribution of the additional burden of tobacco expenditures is also sensitive to the magnitude of the change in price. Larger price changes result in changes in the share of tobacco in household expenditures that are more pro-poor among the poorest 2 quintiles, but they remain less of a burden in terms of relative expenditures in the upper tail of the distribution (Fig. 1).

The difference between total and OOP savings on healthcare is in the form of savings accruing to the health system. These savings, along with the additional tax revenue (which is estimated to be of very large magnitude), give fiscal authorities a lot of room to correct any adverse distributional effects of additional expenditures on tobacco, particularly on continuing smokers in the lowest quintiles.

Distributional considerations provide a more nuanced understanding of how tobacco taxation affects a population and should therefore be taken into account in any tobacco control policy. The results in this paper show that when several outcomes are considered collectively, raising taxes on tobacco would have several pro-poor results.

Nevertheless, we rely on a series of assumptions. Our estimates of health gains are a conservative lower bound because of five simplifying assumptions: i) we only consider the health gains that accrue to quitters, and we exclude health gains that come from a reduction in the intensity of smoking for continuing smokers; ii) we underestimate quitters as we calculate them based only on the elasticity of demand for imported cigarettes and ignore, in this calculation, the other two tobacco goods we consider, which have far more elastic demand; iii) we exclude health gains from reduced exposure to second-hand smoke; iv) we only look at deaths averted and do not take into account other improvements in health; and v) we assume that all quintiles have similar age compositions, and that quintile differences in smoking prevalence are similar for each age cohort, when in fact the differences are likely driven by a higher concentration of younger age cohorts in poorer quintiles, which would mean larger health benefits than we estimate. Our estimates of the related savings on health care spending are also conservative since we also only look at hospitalization costs, only for deaths averted and only for the five disease groups considered. We ignore other health spending and other cases of health gains.

The price measures recommended in the Framework Convention on Tobacco Control and the tax share of price advocated by the World Health Organization all point to the health and public revenue benefits of increasing taxes on tobacco products. In this study, we look further into the distribution of these two benefits and show that raising taxes on tobacco is pro-poor in health gains and the resulting poverty cases averted. We also find that the sheer magnitude of additional tax revenues in addition to the health system savings in the case of Lebanon are larger than any loss from the burden of additional relative spending on tobacco products that poorer continuing smokers may suffer from as a result of the tax, and these added tax revenues would therefore be sufficient to compensate poorer consumers through cessation programs, assistance in health care spending or other cash assistance policies targeted at smokers in the lowest quintiles.

## Figures and Tables

**Table 1:** Inputs used for the modeling of the increase in tobacco excise tax in Lebanon.

Input	Values				Data sources
Smoking prevalence by age (%)			Male	Female	GYTS Global Youth Tobacco Survey Country Factsheet for Lebanon (2011) Sibai and Hwalla (2010) IHME (data for the year 2010); Authors' imputation based on GYTS and Sibai and Hwalla (2010)
		Under 15	18	6	
		15–19	27	13	
		20–24	38	19	
		25–34	41	18	
		35–44	49	33	
		45–54	55	46	
		55–64	46	45	
		65–69	29	21	
		70–74	31	33	
		75–79	25	24	
		80–84	18	19	
		85+	18	19	

Quintile annual household expenditures (2012 USD) per adult equivalent and smoking prevalence rates			Expenditures	Prevalence	Household living conditions survey (2005), inflation (World Development Indicators), National Household Health Expenditures and Use Survey (NHHEUS) 1999 (data for 1996)
		Q1	$1604	28	
		Q2	$2589	28	
		Q3	$3557	27	
		Q4	$4943	25	
		Q5	$9329	22	

Imported cigarettes (in millions of 2012 USD)		339			Customs data, 2012

Expenditures on imported cigarettes by quintile (millions of 2012 USD)		Q1	104		Authors' calculations based on: data on total value of imported cigarettes in 2012 (customs data); share of each quintile in spending by product calculated using 2005 household survey data (CAS, 2005).
		Q2	151		
		Q3	163		
		Q4	180		
		Q5	196		
		Total	794		

Price of imported cigarettes (per pack, 2012 USD)		$2.15			

Share of tax in price, imported cigarettes		47%			Authors' calculations based on Ministry of Finance data (Mahdi, 2014)

Distribution of tobacco-related disease mortality, by cause (%)		COPD	6%		Global Burden of Disease study (IHME, 2013)
		Lung cancer	13%		
		Stroke	19%		
		Ischemic heart disease	55%		
		Hypertensive heart disease	3%		
		Bladder cancer	3%		

Reduction in mortality risk by age at quitting smoking		15–24	98%		Doll et al., 2004
		25–44	85%		
		45–64	75%		
		65+	25%		

Utilization rates of healthcare services by tobacco-related disease		Hypertensive	21%		Authors' calculations (detailed in the data appendix)
		Ischemic	43%		
		Cerebrovascular	29%		
		Respiratory neoplasms	49%		
		Urinary neoplasms	7%		
		Respiratory	26%		

Utilization rates of healthcare services conditional on reporting a health problem (standardized to use Quintile 3 as a reference)		Q1	0.95		Authors' calculations based on NHHEUS 1996
		Q2	0.95		
		Q3	1		
		Q4	1.01		
		Q5	1.08		

Hospitalization cost by tobacco-related disease (2012 USD)		COPD	$951		National Social Security Fund (NSSF) data in Karam (2014). NSSF data is categorized as “Cardiovascular”, “Neoplasm” or “Other”.
		Lung cancer	$2227		
		Stroke	$951		
		Ischemic	$1466		
		Hypertensive	$1466		
		Bladder cancer	$2227		

Fraction of healthcare costs paid out-of-pocket by quintile		Q1	83%		Coverage rates from Salti et al. (2010), including reimbursement rates
		Q2	70%		
		Q3	60%		
		Q4	49%		
		Q5	35%		

Poverty line of expenditures (2012 USD) per person per day		$4			International poverty center, 2008

Poverty rate		29%			International poverty center, 2008

COPD, chronic obstructive pulmonary disease.

**Table 2:** Results from application of the Almost Ideal Demand System (AIDS) model.

Price elasticity of demand for imported tobacco by income quintile (95% confidence interval)		
Q1 (poorest)	−0.32	(−0.47 −0.18)
Q2	−0.27	(−0.36 −0.17)
Q3	−0.26	(−0.40 −0.12)
Q4	−0.24	(−0.34 −0.14)
Q5 (richest)	−0.22	(−0.31 −0.14)

**Table 3:** The impact of a 50% increase in the price of imported cigarettes on health, spending and tax revenues (95% confidence interval).

	Q1 (poorest)	Q2	Q3	Q4	Q5 (richest)	Total
Premature deaths averted	17,000 (9400–24,600)	14,400 (9000–19,000)	13,300 (6100–21,000)	11,000 (6500–16,000)	9000 (6000–13,000)	65,000 (37,000–93,000)
Additional excise tax revenues in millions of USD	36 (28–43)	56 (49–63)	60 (50–72)	69 (60–78)	77 (69–85)	300 (256–341)
% of total borne by quintile	12.0%	18.6%	20.4%	23.1%	25.8%	
% of household expenditures/adult equivalent	2.8%	2.7%	2.1%	1.7%	1.0%	1.7%
Change in expenditures on tobacco products (in millions of USD)	27 (15–38)	45 (35–56)	50 (33–67)	58 (44–71)	66 (52–77)	245 (179–310)
% of household expenditures/adult equivalent	2.1% (1.2%–2.9%)	2.1% (1.7%–2.7%)	1.7% (1.1%–2.3%)	1.4% (1.1%–1.8%)	0.9% (0.7%–1.0%)	1.4% (1.0%–1.7%)
Expenditures on tobacco-related disease treatment averted (in millions of USD)	9 (5–13)	8 (5–10)	8 (4–12)	6 (4–8)	5 (4–8)	37 (21–53)
Out-of-pocket expenditures averted by households (in millions of USD)	8 (4–11)	5 (3–7)	4 (2–7)	3 (2–4)	2 (1–3)	22 (13–33)
% of all savings accruing to Q	36%	23%	18%	14%	9%	
% of household expenditures/adult equivalent	0.60% (0.30–0.86%)	0.20% (0.16–0.35%)	0.10% (0.07–0.24%)	0.07% (0.05–0.11%)	0.02% (0.01–0.04%)	0.10% (0.07–0.18%)
Poverty cases averted	17,000 (9400–24,600)	9800 (3600–7600)	0	0	0	26,800 (13,000–32,200)
Fraction of Q moving out of poverty	2.0%	1.2%	–	–	–	–
