# Supplementary material for: The health, financial and distributional consequences of increases in the tobacco excise tax among smokers in Lebanon
Source: Soc Sci Med. 2016 Dec;170:161–9. doi: 10.1016/j.socscimed.2016.10.020 (PMC5115647; doi:10.1016/j.socscimed.2016.10.020)
Supplement: Supplementary file 1 [file mmc1.docx]

**Table of Contents**

**Appendix 1: Technical Data Appendix**

**Appendix 2: Age-Invariant Elasticities**

**Appendix 3: Participation Elasticity**

**Appendix 4: Extension of Results to Three Tobacco Products and Different Tax Increases**

**Appendix 5: Life Years Gained**

**Appendix 6: Discounting Health Care Savings**

**Appendix 7: Almost Ideal Demand System (AIDS) Model Regressions**

**Appendix 1: Technical Data Appendix**

Utilization rates by diagnosis reported in Table 1 are calculated by comparing *hospitalizations* as reported by the Ministry of Public Health Statistical Report (Ministry of Public Health 2011) and *prevalence rates* of the 5 disease categories of interest. Details on the estimation of these two measures are provided below.

*Hospitalizations*:

The Ministry of Public Health Statistical Report (Ministry of Public Health 2011) includes ministry-covered hospitalizations by diagnosis. We estimate total hospitalizations by assuming MOPH covers 12% of hospitalizations (UNDP 1997).

*Prevalences by diagnosis:*

1. prevalence for hypertension is estimated using WHO database NCD for Lebanon 2009 (WHO 2014a).
2. prevalence for ischemic heart disease is estimated using National Household Health Expenditures and Use Survey (NHHEUS 1999).
3. prevalence for cerebrovascular disease for people 30 and above is found in Jurjus et al (2009).
4. prevalence of lung cancer is imputed from the share of lung cancer patients that die per year in the US (American Lung Association), the share lung cancer’s share in total deaths (IHME 2013), and total deaths (MOPH 2011). The number of hospitalizations per lung cancer patient estimated at 3.3 according to Schneider et al (2007).
5. prevalence for bladder cancer calculated based on the share of bladder cancer patients who die each year in the US (American Cancer Society), the share of bladder cancer deaths in total deaths (IHME 2013), and total deaths (MOPH 2011). The number of hospitalizations per bladder cancer patient estimated at 2.7 by Schneider et al (2007).

**Appendix 2: Age-Invariant Elasticities**

In Table A4 we reproduce the result assuming there is no difference in elasticity between younger and older consumers.

Table A4: Age-invariant elasticities

|  | | | | | | |
| --- | --- | --- | --- | --- | --- | --- |
|  | Q1 (poorest) | Q2 | Q3 | Q4 | Q5 (richest) | Total |
| Premature deaths averted | 13,500 | 11,600 | 10,800 | 9,000 | 7,400 | 52,300 |
|  | (7,600-19,800) | (7,300-15,500) | (5,000-16,500) | (5,300-12,800) | (4,700-10,500) | (29,900-75,100) |
| Expenditures on tobacco-related disease treatment averted (in millions of USD) | 7 | 6 | 6 | 5 | 6 | 30 |
|  | (4-11) | (4-8) | (9-12) | (7-8) | (6-8) | (17-47) |
| Out-of-pocket expenditures averted by households  (in millions of USD) | 6 | 4 | 4 | 3 | 2 | 19 |
|  | (3-9) | (3-6) | (2-6) | (1-4) | (1-2) | (10-27) |
| % of all savings accruing to Q | 31% | 21% | 21% | 16% | 4% |  |
| % of household expenditures/adult equivalent | 0.50% | 0.20% | 0.10% | 0.10% | 0.02% | 0.10% |
|  | (0.3-0.7%) | (0.1-0.3%) | (0.1-0.2%) | (0.04-0.1%) | (0.01-0.03%) | (0.06-0.1%) |
| Poverty cases averted | 13,500 | 4,600 | 0 | 0 | 0 | 18,100 |
|  | (7,600-19,800) | (2,900-6,200) |  |  |  | (10,500-26,000) |
| Fraction of Q moving out of poverty | 1.7% | 0.6% | - | - | - | - |

**Appendix 3: Participation Elasticity**

Appendix Figure A1: Deaths averted for different assumptions about the share of participation in total elasticity of demand for cigarettes

**Appendix 4: Extension of Results to Three Tobacco Products and Different Tax Increases**

This section considers two extensions to the main findings: in a first instance, we look at two additional products in investigating the effect of an increase in tobacco taxes: locally produced cigarettes and waterpipe tobacco. In addition to own-price elasticities, the AIDS model now produces cross-price elasticities across tobacco products. As discussed in the introduction, these two additional tobacco products constitute a relatively small share of total market spending on cigarette products (7% for local cigarettes, 4% for water pipe). The current price of a pack of local cigarettes is estimated at $0.60, a pack of water pipe tobacco at $2.30. However, because there may be substitutions among tobacco products in response to the price change, we consider the effect of a 50% increase in the price of tobacco products across all three tobacco products.

In a second exercise of checking the robustness of the results, different scenarios are considered for the price increase.

*Three tobacco products*

When we consider the consumption of local cigarettes and water-pipe tobacco in addition to imported cigarettes, and include any possible substitutions among the three tobacco products, we note a few important trends. All own-price elasticities are negative, and demand for each of these products is monotonically less sensitive to price as we move to a richer quintile (Table A1). In our analysis, the demand for local cigarettes and for water pipe tobacco is more than unitary elastic for all quintiles.

[Insert table A1 here]

The cross-price elasticities also show that for most pairs of tobacco goods, the cross-price elasticities are positive for all quintiles. This suggests that the products are substitutes. The only exception is in the case of local cigarettes and water pipe tobacco for the poorest quintile. These products have a negative elasticity, suggesting that the poorest quintile consumes these goods together.

In Table A2, we consider the effects of the price increase on household tobacco expenditures and tobacco tax revenue for three scenarios and all three tobacco products, accounting for substitution between tobacco products. The base case considers a 50% increase in the price as was shown for the main results. Overall spending on tobacco products increases by $370 million. The increase in expenditures is a larger fraction of the average household expenditure by adult equivalent of the poorest quintile (4%) than for the richer one (1%).

[Insert Table A2 here]

We also report the effect of potential substitutions on tax revenues. Overall tax revenues almost double as a result of the tax increase, rising by $405 million. Only 14% of the additional tax revenue is financed by the poorest quintile, while 25% is levied from the richest. The additional taxes amount to close to 4% of household expenditures per adult equivalent for households from the poorest quintile, and around 1% for the richest.

With a more conservative price increase of 25%, we find that tax revenues from tobacco rise by $206 million, an increase close to 50%. The additional tax bill is only a slightly larger fraction of the poorest quintile’s average household expenditures per adult equivalent (at 2%) than for the richest (1%). The poorest quintile’s share of the total tax payment is smallest at 12%, and that of the richest quintile is largest at 26%.

With the larger price increase (100%), tax revenues rise by nearly $773 million, close to 190% of current revenues. As a fraction of average household expenditures per adult equivalent, additional tax payments are 9% for the poorest quintile and 2% for the richest. The poorest quintile finances 16% of the additional tax revenues, compared to the richest financing 24%.

Appendix Table A1: Sensitivity analysis results with possible substitution across tobacco products and with different price changes

| **Own- and cross-price elasticities by quintile** |
| --- |
| \|  \|  \| Local \| Imported \| Waterpipe \| \| --- \| --- \| --- \| --- \| --- \| \| *Q1 (poorest)* \|  \|  \|  \|  \| \|  \| Local \| -2.31 \|  \|  \| \|  \| Imported \| 0.22 \| -0.32 \|  \| \|  \| Waterpipe \| -0.78 \| 0.23 \| -1.94 \| \| *Q2* \|  \|  \|  \|  \| \|  \| Local \| -2.3 \|  \|  \| \|  \| Imported \| 0.21 \| -0.27 \|  \| \|  \| Waterpipe \| 0.53 \| 0.09 \| -1.77 \| \| *Q3* \|  \|  \|  \|  \| \|  \| Local \| -1.9 \|  \|  \| \|  \| Imported \| 0.17 \| -0.26 \|  \| \|  \| Waterpipe \| 0.04 \| 0.13 \| -1.73 \| \| *Q4* \|  \|  \|  \|  \| \|  \| Local \| -1.38 \|  \|  \| \|  \| Imported \| 0.14 \| -0.24 \|  \| \|  \| Waterpipe \| 0.21 \| 0.1 \| -1.56 \| \| *Q5 (richest)* \|  \|  \|  \|  \| \|  \| Local \| -1.1 \|  \|  \| \|  \| Imported \| 0.11 \| -0.22 \|  \| \|  \| Waterpipe \| 0.19 \| 0.09 \| -1.39 \|   Full regression results from the AIDS model available upon request from the authors. |

Appendix Table A2. Sensitivity analysis results for different scenarios of price changes and taking substitution across tobacco products into account

| **Effect of a 50% increase in the price of tobacco products on tax revenues and household expenditures on tobacco** |
| --- |
| \|  \| Q1 (poorest) \| Q2 \| Q3 \| Q4 \| Q5 (richest) \| Total \| \| --- \| --- \| --- \| --- \| --- \| --- \| --- \| \| Additional tax revenues \| 56 \| 77 \| 81 \| 91 \| 101 \| 405 \| \| % of total borne by quintile \| 14% \| 19% \| 20% \| 22% \| 25% \|  \| \| % of household expenditures/adult equivalent \| 4.3% \| 3.7% \| 2.8% \| 2.3% \| 1.3% \| 2.3% \| \| Change in expenditures on tobacco products \| 52 \| 73 \| 73 \| 83 \| 90 \| 370 \| \| % of household expenditures/adult equivalent \| 4.0% \| 3.5% \| 2.5% \| 2.1% \| 1.2% \| 2.1% \| |

| **Effect of a 25% increase in the price of tobacco products on tax revenues and household expenditures on tobacco** |
| --- |
| \|  \| Q1 (poorest) \| Q2 \| Q3 \| Q4 \| Q5 (richest) \| Total \| \| --- \| --- \| --- \| --- \| --- \| --- \| --- \| \| Additional tax revenues \| 25 \| 39 \| 42 \| 47 \| 53 \| 206 \| \| % of total borne by quintile \| 12% \| 19% \| 20% \| 23% \| 26% \|  \| \| % of household expenditures/adult equivalent \| 2% \| 2% \| 1% \| 1% \| 1% \| 1% \| \| Change in expenditures on tobacco products \| 20 \| 37 \| 38 \| 43 \| 47 \| 185 \| \| % of household expenditures/adult equivalent \| 2% \| 2% \| 1% \| 1% \| 1% \| 1% \| |
| **Effect of a 100% increase in the price of tobacco products on tax revenues and household expenditures on tobacco** |
| \|  \| Q1 (poorest) \| Q2 \| Q3 \| Q4 \| Q5 (richest) \| Total \| \| --- \| --- \| --- \| --- \| --- \| --- \| --- \| \| Additional tax revenues \| 121 \| 149 \| 150 \| 170 \| 183 \| 773 \| \| % of total borne by quintile \| 16% \| 19% \| 19% \| 22% \| 24% \|  \| \| % of household expenditures/adult equivalent \| 9% \| 7% \| 5% \| 4% \| 2% \| 4% \| \| Change in expenditures on tobacco products \| 121 \| 141 \| 134 \| 154 \| 160 \| 710 \| \| % of household expenditures/adult equivalent \| 9% \| 7% \| 5% \| 4% \| 2% \| 4% \| |

**Appendix 5: Life Years Gained**

We estimate years of life gained for quitters over the 60 years following the tax increase which results in a 50% increase in the price of tobacco products, and more specifically in the price of imported cigarettes. To calculate years of life gained from an increase in the price of cigarettes, we assume, as we do in the calculation of deaths averted, that half of the price elasticity of demand estimated is an elasticity of participation. We also assume everyone dies when they reach an age equal to life expectancy, which in Lebanon is 74. So for each cohort, the years of life are gained by quitting accrue at the time the cohort turns 74.

Doll et al (2004) find that quitters gain 10, 9, 6 and 3 years of life if they quit smoking at age 30, 40, 50 or 60, respectively. Based on these four values of quitting age, we impute the expected years of life gained for quitters of other ages by fitting years of life gained as a linear function of the log of the difference between life expectancy and age at quitting:

where *LE* is life expectancy and *age* is the age at quitting.

The results show an overall gain of 690,385 life years over 60 years. This amounts to the length of close to 9,329 lives given current life expectancy. This gain in life years is distributed progressively:

Years of life gained by quintile

Appendix Table A3: Years of life gained (YLG).

| Quintile | Q1 (poorest) | Q2 | Q3 | Q4 | Q5 (Richest) | Total |
| --- | --- | --- | --- | --- | --- | --- |
| YLG | 177623 | 152697 | 141596 | 119393 | 99075 | 690385 |

**Appendix 6: Discounting Health Care Savings**

Appendix Table A5: Net present value of heath care savings using a 3% annual discount rate

|  | | | | | | |
| --- | --- | --- | --- | --- | --- | --- |
|  | Q1 (poorest) | Q2 | Q3 | Q4 | Q5 (richest) | Total |
| Expenditures on tobacco-related disease treatment averted (in millions of USD) | 3.5 | 3 | 3 | 2.5 | 2.2 | 14.2 |
|  | (2.0-13) | (1.9-10) | (1.4-12) | (1.5-8) | (1.4-8) | (8.2-53) |
| Out-of-pocket expenditures averted by households  (in millions of USD) | 3 | 2.1 | 1.8 | 1.2 | 0.8 | 8.9 |
|  | (1.7-5.2) | (1.3-4.1) | (0.8-4.6) | (0.7-3.6) | (0.5-3.1) | (5-20.6) |
| % of all savings accruing to Q | 34% | 24% | 20% | 13% | 9% |  |
| % of household expenditures/adult equivalent | 0.22% | 0.10% | 0.06% | 0.03% | 0.01% | 0.05% |
|  | (0.13-0.34%) | (0.06-0.14%) | (0.03-0.09%) | (0.02-0.04%) | (0.01-0.01%) | (0.03-0.07%) |

**Appendix 7: AIDS Model Regressions**

This appendix includes the results of the AIDS model regressions. The elasticities are calculated using coefficients from the seemingly unrelated regressions reported below, based on the AIDS model constraints. The following regressions report the Heckman selection model regressions for households that consume any local cigarettes and any imported cigarettes.

The variable labels are:

Local cig: log price of local cigarettes

Imported cig: log price of imported cigarettes

Hookah tob: log price of hookah tobacco

Hh size: household hize

Hh size squared: the square of household size

Share of children <6 y. o.: share of household members under 6 years of age

Share of children 7-14 y. o.: share of household members between ages 7 and 14

Share of children 15-18 y. o.: share of household members between 15 and 18

Share of adult males: share of household members who are adult males

Share of adult females: share of household members who are adult females

Share of elderly: share of household members above 65

Share unemployed: share of household members unemployed

Share job holders: share of household members who hold a job

Share primary ed: share of household members who have a primary education

Share univ: share of household members who hold a university degree

Female headed: indicator variable that equals 1 if the household head is a female, 0 otherwise

Head age: age of household head

Head single: indicator variable that equals 1 if the household head is single, 0 otherwise

Rooms: total number of rooms in the house

Cars: total number of cars owned by the household

Disability: indicator variable that equals 1 if at least one member of the household is disabled, 0 otherwise

All insured: indicator variable that equals 1 if all members of the household have health insurance coverage, 0 otherwise

Has maid: indicator variable that equals 1 if the household employs a maid, 0 otherwise

Income: household income per adult equivalent

Appendix Table A6: Seemingly Unrelated Regressions of the Share of Tobacco Expenditures on Local Cigarettes and Imported Cigarettes

|  | Quintile 1 (poorest) | | Quintile 2 | | Quintile 3 | | Quintile 4 | | Quintile 5 (richest) | |
| --- | --- | --- | --- | --- | --- | --- | --- | --- | --- | --- |
|  | Local cig | Imp. cig | Local cig | Imp. cig | Local cig | Imp. cig | Local cig | Imp. cig | Local cig | Imp. cig |
| *Log price* |  |  |  |  |  |  |  |  |  |  |
| Local cig | -.12**  (.02) | .11**  (.02) | -.12**  (.01) | .10**  (.01) | -.08**  (.01) | .07**  (.02) | -.04**  (.01) | .05**  (.01) | -.02**  (.004) | .02**  (.01) |
| Imported cig | .11**  (.02) | -.14*  (.06) | .10**  (.01) | -.10*  (.04) | .07**  (.02) | -.09  (.06) | .04**  (.01) | -.08^+^  (.04) | .02**  (.01) | -.06  (.04) |
| Hookah tob | -.07**  (.03) | .14**  (.04) | .03*  (.02) | .02  (.02) | -.002  (.01) | .06**  (.02) | .01  (.01) | .03^+^  (.02) | .01  (.01) | .02  (.02) |
| *Household characteristics* |  |  |  |  |  |  |  |  |  |  |
| Hh size | -.03  (.03) | -.1**  (.03) | .003  (.02) | -.04  (.03) | .02  (.02) | .01  (.02) | -.01  (.01) | .02  (.02) | -.02*  (.01) | .03  (.02) |
| Hh size squared | .002  (.003) | .01*  (.003) | .001  (.001) | .001  (.002) | -.0002  (.001) | .001  (.002) | .001  (.001) | -.001  (.001) | .002*  (.001) | -.003*  (.001) |
| Share of children <6 y. o. | 1.68**  (.55) | -.74  (.65) | -.28  (.36) | 1.03^+^  (.57) | -.19*  (.08) | .15^+^  (.1) | .04  (.06) | .08  (.08) | -.02  (.04) | -.02  (.07) |
| Share of children 7-14 y. o. | 1.79**  (.56) | -.69  (.65) | -.25  (.36) | 1.08^+^  (.56) | -.10  (.08) | .11  (.1) | .06  (.06) | .08  (.09) | -.01  (.04) | -.01  (.08) |
| Share of children 15-18 y. o. | 1.63**  (.55) | -.97  (.65) | -.08  (.36) | .63  (.57) |  |  |  |  |  |  |
| Share of adult males | 1.59**  (.53) | -.93  (.64) | -.29  (.36) | .88  (.56) | -.13^+^  (.08) | .20^+^  (.11) | .04  (.07) | .02  (.09) | -.02  (.04) | .04  (.07) |
| Share of adult females | 1.61**  (.54) | -.80  (.63) | -.23  (.36) | .91  (.56) | -.06  (.08) | .10  (.11) | -.02  (.06) | .15^+^  (.09) | -.04  (.04) | .05  (.07) |
| Share of elderly | 1.74**  (.54) | -.91  (.64) | -.23  (.36) | .97+  (.56)) | -.11  (.08) | .16  (.10) | -.004  (.06) | .07  (.09) | -.03  (.04) | .05  (.07) |
| Share unempl | .17^+^  (.09) | -.05  (.10) | -.07  (.07) | .20*  (.09) | .07  (.07) | -.07  (.09) | -.03  *.06) | .11  (.08) | .03  (.04) | -.08  (.08) |
| Disability | .04  (.04) | -.002  (.05) | -.002  (.03) | -.01  (.04) | .01  (.03) | -.003  (.03) | -.02  (.02) | .03  (.03) | -.01  (.02) | .02  (.03) |
| All insured | -.07*  (.03) | .01  (.04) | -.03  (.02) | .03  (.03) | -.01  (.02) | .02  (.02) | -.03**  (.01) | .04*  (.02) | -.02  (.01) | .02  (.01) |
| Has maid |  |  |  | .21  (.19) | -.18  (.19) | .18  (.16) | .02  (.04) | .02  (.06) | -.01  (.01) | .006  (.03) |
| Income | -.06*  (.03) | .11**  (.04) | .03  (.02) | .0001  (.03) | -.02  (.02) | .06  (.04) | .01  (.02) | .02  (.03) | .01  (.01) | .002^+^  (.02) |
| Mill’s ratio  (local cig) | -.12**  (.04) |  | -.04*  (.02) |  | .01  (.03) |  | -.02  (.02) |  | -.01  (.01) |  |
| Mill’s ratio (imported cig) |  | -.16**  (.05) |  | -.14*  (.07) |  | -.01  (.06) |  | -.01 |  | .02  (.05) |
| Constant |  |  |  |  | .43  (.39) | -.26  (.67) | -.001  (.32) | .45 | -.003  (.15) | .77**  (.28) |
| observations | 742 | 742 | 968 | 968 | 1032 | 1032 | 1034 | 1034 | 1045 | 1045 |

Notes: Standard errors in parentheses. ** significant at the 1% level, * significant at the 5% level, + significant at the 10% level. For each quintile, dependent variables are share of total tobacco spending on local (first column) and imported (second column) cigarettes.

Appendix Table A7: Heckman Selection Models for Quintile 1

|  |  |  |  |  |
| --- | --- | --- | --- | --- |
|  | Share local | Participation | Share imp | Participation |
|  | (1) | (2) | (3) | (4) |
| *Log price* |  |  |  |  |
| Local cig | .10**  (.02) |  |  |  |
| Imported |  |  | -.001  (.004) |  |
| *Household characteristics* |  |  |  |  |
| Hh size | -.32**  (.09) | .73**  (.11) | -.06**  (.02) | .47**  (.08) |
| Hh size squared | .02**  (.01) | -.05**  (.01) | .0.4**  (.001) | -.03**  (.01) |
| Share of children <6 | .39^+^  (.21) | .29  (.41) | .15**  (.05) | .39  (.33) |
| Share of children 7-14 | .89**  (.28) | 1.97**  (.54) | .12*  (.06) |  |
| Share of children 15-18 | -.36  (.24) | 1.85**  (.43) | -.11  (.07) | 1.2**  (.46) |
| Share of adult males | -.18  (.12) | 1.85**  (.42) | .003  (.03) | 1.36**  (.34) |
| Share of adult females | .05  (.15) | 1.67**  (.42) | .03  (.03) | 1.11**  (.34) |
| Share of elderly |  | 2.20**  (.49) |  | .81*  (.39) |
| Share job holders |  | .55**  (.21) |  | .10  (.15) |
| Share primary ed |  | -.03  (.16) |  | .01  (.12) |
| Share univ |  | -.28  (.71) |  | -.25  (.53) |
| Disability |  | .05  (.15) |  | -.27*  (.13) |
| All insured |  | .04  (.11) |  | -.005  (.09) |
| Rooms |  | .04  (.05) |  | -.001  (.04) |
| Cars |  | .02  (.12) |  | .28**  (.10) |
| *Head characteristics* |  |  |  |  |
| Female headed |  | -.15  (.13) |  | -.11  (.10) |
| Head age |  | -.01*  (.005) |  | -.002  (.004) |
| Head Univ |  | -.09  (.48) |  | -.35  (.41) |
| Head single |  | -.22  (.19) |  | .12  (.13) |
| Mills Lambda |  | -.41**  (.11) |  | -.05  (.03) |
| Rho |  | -.79 |  | -.29 |
| Sigma |  | .51 |  | .17 |
| Observations | 1477 | | 1477 | |
| Censored | 1233 | | 904 | |
| Wald chi2 (8) | 61.67 | | 34.99 | |

Notes: Standard errors in parentheses. ** significant at the 1% level, * significant at the 5% level, + significant at the 10% level. Dependent variables are: share of tobacco spending on local cigarettes in column (1), the presence of any tobacco spending on local cigarettes in column (2), share of tobacco spending on imported cigarettes in column (3) and the presence of any spending on local cigarettes in column (4). Selection regressions (2) and (4) also include controls for governorate and an intercept (not shown here).Appendix Table A8: Heckman Selection Models for Quintile 2

|  | Share local | Participation | Share imp | Participation |
| --- | --- | --- | --- | --- |
|  | (1) | (2) | (3) | (4) |
| *Log price* |  |  |  |  |
| Local cig | .21**  (.02) |  |  |  |
| Imported |  |  | -.0002  (.009) |  |
| *Household characteristics* |  |  |  |  |
| Hh size | .09  (.05) | .34**  (.1) | -.04**  (.01) | .37**  (.08) |
| Hh size squared | -.004  (.004) | -.01^+^  (.01) | .0.02*  (.001) | -.02**  (.01) |
| Share of children <6 | -.15  (.18) | -.92^+^  (.51) | .11**  (.03) | -.09  (.39) |
| Share of children 7-14 | -.10  (.2) | -.95*  (.47) | .10**  (.04) | -.22  (.36) |
| Share of children 15-18 | .23  (.25) | .91^+^  (.52) | -.16**  (.05) | -.24  (.44) |
| Share of adult males | -.20  (.16) | .09  (.36) | -.01  (.03) | .61*  (.27) |
| Share of adult females | -.11  (.19) | .04  (.33) | .01  (.03) | .14  (.24) |
| Share job holders |  | .38  (.25) |  | .29  (.19) |
| Share primary ed |  | -.09  (.17) |  | .36**  (.13) |
| Share univ |  | 1.43**  (.53) |  | .35  (.44) |
| Disability |  | -.19  (.16) |  | -.14  (.13) |
| All insured |  | -.05  (.11) |  | .03  (.08) |
| Has maid |  |  |  | -.35  (.50) |
| Rooms |  | -.08^+^  (.04) |  | -.03  (.03) |
| Cars |  | -.15^+^  (.09) |  | .05  (.07) |
| *Head characteristics* |  |  |  |  |
| Female headed |  | -.19  (.15) |  | -.22^+^  (.11) |
| Head age |  | .003  (.005) |  | .002  (.004) |
| Head Univ |  | -.91*  (.42) |  | -.33  (.29) |
| Head single |  | -.58^+^  (.30) |  | .06  (.17) |
| Mills Lambda |  | -.34**  (.98) |  | -.02  (.03) |
| Rho |  | .74 |  | -.17 |
| Sigma |  | .45 |  | .14 |
| Observations | 1479 | | 1479 | |
| Censored | 1222 | | 632 | |
| Wald chi2 (8) | 84.8 | | 63.81 | |

Notes: Standard errors in parentheses. ** significant at the 1% level, * significant at the 5% level, + significant at the 10% level. Dependent variables are: share of tobacco spending on local cigarettes in column (1), the presence of any tobacco spending on local cigarettes in column (2), share of tobacco spending on imported cigarettes in column (3) and the presence of any spending on local cigarettes in column (4). Selection regressions (2) and (4) also include controls for governorate and an intercept (not shown here).Appendix Table A9: Heckman Selection Models for Quintile 3

|  | Share local | Participation | Share imp | Participation |
| --- | --- | --- | --- | --- |
|  | (1) | (2) | (3) | (4) |
| *Log price* |  |  |  |  |
| Local cig | -.19**  (.03) |  |  |  |
| Imported |  |  | .02**  (.01) |  |
| *Household characteristics* |  |  |  |  |
| Hh size | .19**  (.06) | .28**  (.09) | -.001  (.01) | .37**  (.08) |
| Hh size squared | -.006  (.004) | -.006  (.007) | -.0.001  (.001) | -.02**  (.01) |
| Share of children <6 | -.77**  (.28) | -1.30*  (.52) | .07*  (.03) | -.37  (.39) |
| Share of children 7-14 | -.17  (.28) | -1.14*  (.46) | .07*  (.03) | -.04  (.36) |
| Share of children 15-18 | .10  (.31) | -.10  (.51) | -.07  (.05) | -.18  (.43) |
| Share of adult males | -.15  (.21) | .05  (.35) | .004  (.03) | 1.08**  (.28) |
| Share of adult females | .06  (.23) | -.03  (.34) | -.01  (.03) | .40  (.26) |
| Share job holders |  | .58*  (.24) |  | .27  (.20) |
| Share primary ed |  | .16  (.17) |  | .44**  (.14) |
| Share univ |  | -.79  (.52) |  | -.07  (.34) |
| Disability |  | .03  (.15) |  | -.28*  (.13) |
| All insured |  | -.10  (.10) |  | -.06  (.08) |
| Has maid |  |  |  | -.60  (.37) |
| Rooms |  | .004  (.04) |  | .03  (.03) |
| Cars |  | .06  (.09) |  | .12  (.07) |
| *Head characteristics* |  |  |  |  |
| Female headed |  | -.08  (.17) |  | .08  (.14) |
| Head age |  | -.005  (.005) |  | -.001  (.004) |
| Head Univ |  | .09  (.29) |  | -.17  (.20) |
| Head single |  | -.12  (.25) |  | -.35^+^  (.19) |
| Mills Lambda |  | .69**  (.15) |  | .02  (.03) |
| Rho |  | 1 |  | -.17 |
| Sigma |  | .69 |  | .14 |
| Observations | 1476 | | 1476 | |
| Censored | 1236 | | 546 | |
| Wald chi2 (8) | 48.27 | | 36.64 | |

Notes: Standard errors in parentheses. ** significant at the 1% level, * significant at the 5% level, + significant at the 10% level. Dependent variables are: share of tobacco spending on local cigarettes in column (1), the presence of any tobacco spending on local cigarettes in column (2), share of tobacco spending on imported cigarettes in column (3) and the presence of any spending on local cigarettes in column (4). Selection regressions (2) and (4) also include controls for governorate and an intercept (not shown here).Appendix Table A10: Heckman Selection Models for Quintile 4

|  |  |  |  |  |
| --- | --- | --- | --- | --- |
|  | Share local | Participation | Share imp | Participation |
|  | (1) | (2) | (3) | (4) |
| *Log price* |  |  |  |  |
| Local cig | -.08**  (.02) |  |  |  |
| Imported |  |  | .008  (.007) |  |
| *Household characteristics* |  |  |  |  |
| Hh size | .06  (.04) | .22**  (.08) | -.01  (.01) | .33**  (.07) |
| Hh size squared | -.001  (.003) | -.005  (.006) | .0005  (.0007) | -.01**  (.005) |
| Share of children <6 | -.08  (.20) | -.15  (.52) | .05  (.04) | -.90*  (.41) |
| Share of children 7-14 | -.05  (.21) | -.48  (.46) | .01  (.03) | -.97**  (.37) |
| Share of children 15-18 | -.33  (.31) | -1.13*  (.57) | .005  (.05) | -.40  (.45) |
| Share of adult males | .11  (.18) | .38  (.37) | .02  (.03) | .28  (.29) |
| Share of adult females | -.14  (.20) | .03  (.37) | -.01  (.03) | -.14  (.29) |
| Share job holders |  | .45^+^  (.24) |  | .17  (.21) |
| Share primary ed |  | .19  (.19) |  | .33*  (.16) |
| Share univ |  | .28  (.37) |  | -.09  (.30) |
| Disability |  | .09  (.15) |  | -.06  (.14) |
| All insured |  | -.23*  (.10) |  | -.02  (.08) |
| Has maid |  |  |  | .06  (.14) |
| Rooms |  | -.02  (.04) |  | .01  (.03) |
| Cars |  | -.18^+^  (.10) |  | -.02  (.09) |
| *Head characteristics* |  |  |  |  |
| Female headed |  | -.26  (.17) |  | -.06  (.14) |
| Head age |  | .001  (.006) |  | -.01*  (.005) |
| Head Univ |  | -.09  (.19) |  | -.07  (.16) |
| Head single |  | -.53^+^  (.27) |  | -.63**  (.20) |
| Mills Lambda |  | .45**  (.09) |  | .01  (.03) |
| Rho |  | .091 |  | .05 |
| Sigma |  | .50 |  | .14 |
| Observations | 1477 | | 1477 | |
| Censored | 1243 | | 501 | |
| Wald chi2 (8) | 18.37 | | 5.57 | |

Notes: Standard errors in parentheses. ** significant at the 1% level, * significant at the 5% level, + significant at the 10% level. Dependent variables are: share of tobacco spending on local cigarettes in column (1), the presence of any tobacco spending on local cigarettes in column (2), share of tobacco spending on imported cigarettes in column (3) and the presence of any spending on local cigarettes in column (4). Selection regressions (2) and (4) also include controls for governorate and an intercept (not shown here).Appendix Table A11: Heckman Selection Models for Quintile 5

|  | Share local | Participation | Share imp | Participation |
| --- | --- | --- | --- | --- |
|  | (1) | (2) | (3) | (4) |
| *Log price* |  |  |  |  |
| Local cig | -.05**  (.01) |  |  |  |
| Imported |  |  | .008+  (.005) |  |
| *Household characteristics* |  |  |  |  |
| Hh size | .005  (.04) | .33**  (.12) | -.004  (.01) | .42**  (.10) |
| Hh size squared | .002  (.003) | -.01  (.01) | 9e-5  (.001) | -.02**  (.01) |
| Share of children <6 | -.04  (.16) |  | -.006  (.04) |  |
| Share of children 7-14 | .04  (.15) | .26  (.43) | .07*  (.03) | .27  (.33) |
| Share of children 15-18 | .09  (.18) | .08  (.47) | .05  (.04) | 1.24**  (.37) |
| Share of adult males | .24+  (.14) | 1.13*  (.45) | .05+  (.03) | 1.11**  (.36) |
| Share of adult females | .08  (.15) | .91*  (.46) | .03  (.03) | .98**  (.37) |
| Share job holders |  | .38  (.25) |  | .54*  (.22) |
| Share primary ed |  | .87**  (.25) |  | .70**  (.16) |
| Share univ |  | .46  (.31) |  | .16  (.27) |
| Disability |  | -.52**  (.20) |  | -.25+  (.15) |
| All insured |  | -.07  (.10) |  | .04  (.08) |
| Has maid |  | .23  (.15) |  | -.46**  (.13) |
| Rooms |  | -.04  (.03) |  | .006  (.03) |
| Cars |  | -.11  (.15) |  | -.10  (.15) |
| *Head characteristics* |  |  |  |  |
| Female headed |  | -.19  (.18) |  | -.40**  (.14) |
| Head age |  | -.01  (.01) |  | -.01  (.005) |
| Head Univ |  | -.26+  (.15) |  | -.10  (.12) |
| Head single |  | -.98**  (.11) |  | -.17  (.25) |
| Mills Lambda |  | .30**  (.06) |  | .05+  (.02) |
| Rho |  | .088 |  | .36 |
| Sigma |  | .33 |  | .13 |
| Observations | 1475 | | 1475 | |
| Censored | 1217 | | 476 | |
| Wald chi2 (8) | 19.38 | | 12.52 | |

Notes: Standard errors in parentheses. ** significant at the 1% level, * significant at the 5% level, + significant at the 10% level. Dependent variables are: share of tobacco spending on local cigarettes in column (1), the presence of any tobacco spending on local cigarettes in column (2), share of tobacco spending on imported cigarettes in column (3) and the presence of any spending on local cigarettes in column (4). Selection regressions (2) and (4) also include controls for governorate and an intercept (not shown here).
